# Selection of target sequences as well as sequence identity determine the outcome of RNAi approach for resistance against cotton leaf curl geminivirus complex

**DOI:** 10.1186/1743-422X-8-122

**Published:** 2011-03-16

**Authors:** Muhammad Mubin, Mazhar Hussain, Rob W Briddon, Shahid Mansoor

**Affiliations:** 1 Agricultural Biotechnology Division, National Institute for Biotechnology and Genetic Engineering, Jhang Road, Faisalabad, Pakistan

## Abstract

Cotton leaf curl disease is caused by a geminivirus complex that involves multiple distinct begomoviruses and a disease-specific DNA satellite, cotton leaf curl Multan betasatellite (CLCuMB), which is essential to induce disease symptoms. Here we have investigated the use of RNA interference (RNAi) for obtaining resistance against one of the viruses, *Cotton leaf curl Multan virus *(CLCuMV), associated with the disease. Three hairpin RNAi constructs were produced containing either complementary-sense genes essential for replication/pathogenicity or non-coding regulatory sequences of CLCuMV. In transient assays all three RNAi constructs significantly reduced the replication of the virus in inoculated tissues. However, only one of the constructs, that targeting the overlapping genes involved in virus replication and pathogenicity (the replication-associated protein (Rep), the transcriptional activator protein and the replication enhancer protein) was able to prevent systemic movement of the virus, although the other constructs significantly reduced the levels of virus in systemic tissues. In the presence of CLCuMB, however, a small number of plants co-inoculated with even the most efficient RNAi construct developed symptoms of virus infection, suggesting that the betasatellite may compromise resistance. Further analyses, using Rep gene sequences of distinct begomoviruses expressed from a PVX vector as the target, are consistent with the idea that the success of the RNAi approach depends on sequence identity to the target virus. The results show that selection of both the target sequence, as well as the levels of identity between the construct and target sequence, determine the outcome of RNAi-based resistance against geminivirus complexes.

## Introduction

Cotton leaf curl is a serious disease of cotton and several other malvaceous plant species that is transmitted by the whitefly *Bemisia tabaci *[1]. The disease is, at this time, endemic throughout Pakistan and western India [2,3]. Affected cotton plants exhibit a range of symptoms that include leaf curling, stunted growth and a poor yield of cotton fibre [1,4]. Additionally, affected plants may develop leaf-like outgrowths from the veins on the underside of leaves. The disease is caused by a geminivirus complex that involves several distinct begomoviruses (genus *Begomovirus*, family *Geminiviridae*) that interact with a disease-specific DNA satellite, Cotton leaf curl Multan betasatellite (CLCuMB) [3,5-7].

The geminiviruses are a rapidly emerging group of plant viruses, which can be attributed to various factors, including increased insect vector populations, the presence of alternate hosts and recombination among viruses [8,9]. Geminiviruses are plant-infecting viruses with circular single-stranded (ss)DNA genomes of 2.5-5.6 kb [10]. Whitefly-transmitted geminiviruses are classified in the genus *Begomovirus *which encompasses many of the agriculturally most destructive geminiviruses. All begomoviruses native to the New World have bipartite genomes, with components known as DNA A and DNA B. In the Old World, although a few bipartite begomoviruses have been identified, the majority of begomoviruses have genomes consisting of a single component, homologous to the DNA A component of the bipartite viruses [11,12]. A small number of truly monopartite begomoviruses have been characterized, such as *Tomato leaf curl virus *and *Tomato yellow leaf curl Sardinia virus *(TYLCSV) [13,14]. However, the majority of monopartite begomoviruses are associated with addition ssDNA components, known as betasatellites and alphasaltellites [15]. The betasatellites are symptom modulating satellites which, in some cases, are required by their helper begomoviruses to systemically infect the plants from which they were isolated; this is the case for the begomoviruses that cause CLCuD, such as *Cotton leaf curl Multan virus *(CLCuMV), which require CLCuMB to efficiently infect cotton and induce *bona fide *disease symptoms [7,16]. The betasatellites encode a single protein which is a pathogenicity (symptom) determinant and a suppressor of RNA interference (RNAi)-based host defenses [17-19]. The alphasatellites are associated with the majority of begomovirus-betasatellite complexes, including that which causes CLCuD [20-22]. They are not essential but recent results suggest that they may be involved in overcoming host defenses [23].

The genomes of monopartite begomoviruses encode six proteins with genes in the virion and complementary-sense separated by a non-coding intergenic (IR) region that contains control sequences as well as the virion-sense origin of replication [24]. The genes in the virion-sense encode the V2 protein, which is involved in virus movement and is a suppressor of RNAi, and the coat protein, the only structural protein of geminiviruses that is required to form the characteristic geminate particles, for movement in plants and interacts with the whitefly vector for transmission plant-to-plant. In the complementary-sense the genes encode the replication associated protein (Rep; the only virus encoded protein required for viral DNA replication, which is a rolling-circle replication-initiator protein), the transcriptional activator protein (TrAP; involved in the up-regulation of late (virion-sense) genes, modulating host gene expression and may be a suppressor of RNAi), the replication-enhancer protein (REn; interacts with and enhances Rep activity) and the C4 protein (a suppressor of RNAi that may be involved in virus movement).

The control of CLCuD, as is the case for most geminivirus diseases, is mainly based on control of the vector using insecticides and the cultivation of resistant crop varieties [8]. Resistant cotton cultivars were introduced in the mid to late 1990 s that were developed by conventional breeding/selection. After initially showing promise in the control of CLCuD, the virus complex ultimately overcame the resistance [25], although the precise changes in the complex responsible remain unclear. Both the virus and the betasatellite associated with resistance breaking have been shown to be recombinant [3,5] and efforts continue to identify the precise molecular basis for their ability to overcome host resistance in cotton. Thus, alternate strategies are required to prevent losses due to the disease. Among the possible strategies, those based on RNAi have shown some promise [26].

RNAi is a ubiquitous phenomenon in eukaryotic organisms that is triggered by double-stranded RNA (dsRNA) that plays important roles in diverse biological processes. The key features of RNA silencing include the production of 21-25 nucleotide small interfering RNAs (siRNA) by enzymes known as Dicers and the formation of RNA-induced silencing complexes (RISCs) which contain Argonaute proteins that directly carry out gene silencing at the transcriptional or posttranscriptional levels [27]. RNA silencing can be activated by introducing transgenes, RNA viruses or DNA sequences that are homologous to expressed genes. This phenomenon has been utilized as a tool to study various molecular processes in the cell, chromosome organization, functions of genes and for obtaining resistance to viruses in plants and animals. As a counter defense viruses have evolved "suppressor" proteins, which are able to prevent or counter RNAi [28].

Several studies investigating the use of RNAi for obtaining resistance against geminiviruses have been reported, with different levels of success (reviewed by [29,30]). However, there are only two reports investigating the use of RNAi against a begomovirus-betasatellite complex [31,32]. Here RNAi has been investigated as a means of obtaining resistance against one of the viruses causing CLCuD.

## The Study

### Production of RNAi constructs

**Figure 1:**
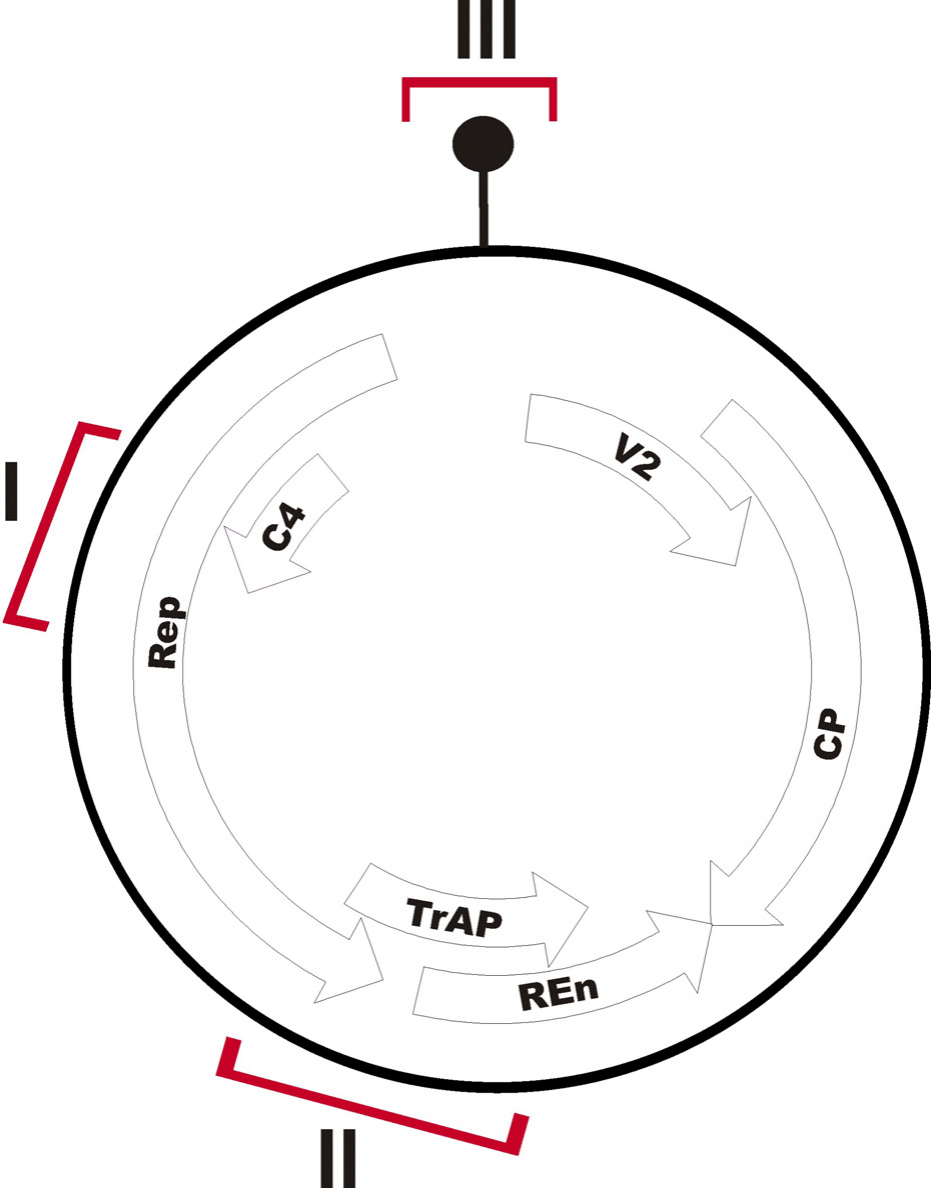
**Sequences of Cotton leaf curl Multan virus (CLCuMV) used to produce hairpin constructs**. The sequences used to produce hairpin constructs are highlighted in red on the genome map of CLCuMV. The virus-encoded genes and their orientation are indicated as arrows. The genes encode the V2 protein, the coat protein (CP), the replication-associated protein (Rep), the transcriptional activator protein (TrAP), the replication enhancer protein (REn) and the C4 protein. The origin of virion-sense DNA replication is indicated by the black dot at position zero. Sequence I consisted of the overlapping Rep and C4 gene sequences which was used to produce construct CLCRNAiRepC4/pFGC. Sequence II consisted of the overlapping Rep, TrAP and REn gene sequences and was used to produce construct CLCRNAiRepTrAPREn/pFGC. Sequence III contained sequence derived from the intergenic region and was used to make construct CLCRNAiIR/pFGC.

Three hairpin gene constructs targeting CLCuMV were produced. These were based on sequences of the Rep and C4 genes (CLCRNAiRepC4/pFGC), the Rep, TrAP and REn genes (CLCRNAiRepTrAPREn/pFGC), and on the IR (CLCRNAiIR/pFGC). The primers for PCR-mediated amplification of the respective sequences were designed to the published sequence of CLCuMV (AJ496287) and are given in Table 1. The positions of the sequences used in the constructs are shown relative to the genome map of CLCuMV in Figure 1. The amplified DNA fragments were cloned in sense and antisense orientations in the pFGC5941 dsRNA binary vector [33] using restriction endonuclease recognition sequences included in the primers (Table 1).

**Table 1:** Primers used for amplification of virus sequences for the production of hairpin RNAi constructs.

**Construct**	**Primer**	Sequence*	**Cloning sites in sense orientation**	**Cloning sites in antisense orientation**
CLCRNAiRepC4/pFGC	AC1-4 SF AC1-4 SR AC1-4 ASF AC1-4 ASR	GTTCCATGGCCCAAACTTCAAGTTCTTC CAGGGCGCGCCCATCAGTTGTCTCCAAT CAAGGATCCCCCCAAACGTTTTAAAGTAC TTCTCTAGAACTTGATCGAAAGAAGAAGG	*Asc*I+*Nco*I	*Bam*HI+*Xba*I
CLCRNAiRepTrAPREn/pFGC	AC123 SF AC123 SR AC123 ASF AC123 ASR	CCCCTCGAGGCTACGTTGAAGATTAGGAT TTCGGCGCGCCTCCTGGTCCCAACAGCAGT TTCTCTAGAAATCCTGGTCCCAACAGCAGT CCCCCCGGGGCTACGTTGAAGATTAGGAT	*Xho*I+*Asc*I	*Xba*I+*Sma*I
CLCRNAiIR/pFGC	IR SF IR SR IR ASF IR ASR	TGACTCGAGTCAATTGGAGACAACTGAT GTTCCATGGTGAAACTTAGTGCGCAAG GTTGGATCCTGAAACTTAGTGCGCAAG TGATCTAGATCAATTGGAGACAACTGAT	*Xho*I+*Nco*I	BamHI+*Xba*I

* The restriction sites included in primers are underlined.

### Transient infectivity assays

*Agrobacterium tumefaciens *strain GV3101 cultures harbouring the pFGC5941 constructs were co-infiltrated to young *N. benthamiana *plants with partial, direct-repeat constructs of CLCuMV and CLCuMB [6]. Replication of the virus was detected by Southern hybridization at 15 days post-infiltration in systemic leaves. A radioactively-labeled, full-length CLCuMV probe was used for hybridization at 65°C overnight followed by two washes with 2× SSC/0.5% SDS and 1× SSC/0.5% SDS at 65°C, as described previously [6].

### Resistance to homologous Rep gene sequences

The resistance to heterologous virus sequences imparted by construct CLCRNAiRepTrAPREn/pFGC was assessed by co-inoculation with *Potato virus X *(PVX) vector constructs expressing the Rep gene sequences of heterologous begomoviruses. The production of PVX vector (pgR107; [34]) constructs for the expression of the Rep genes of CLCuMV, African cassava mosaic virus (ACMV) and Cabbage leaf curl virus (CaLCuV) have been described previously (Imran Amin, Basavaprabhu L. Patil, Rob W. Briddon, Shahid Mansoor and Claude M. Fauquet.

Comparison of phenotypes produced in response to transient expression of genes encoded by four distinct begomoviruses in *Nicotiana benthamiana *and their correlation with the levels of developmental miRNAs, submitted). *A. tumefaciens *strain GV3101 cultures harbouring the CLCRNAiRepTrAPREn/pFGC construct and the PVX vector constructs were co-infiltrated to *N. benthamiana *plants as described above.

## Results

### RNAi constructs for CLCuMV in transient assays

**Figure 2:**
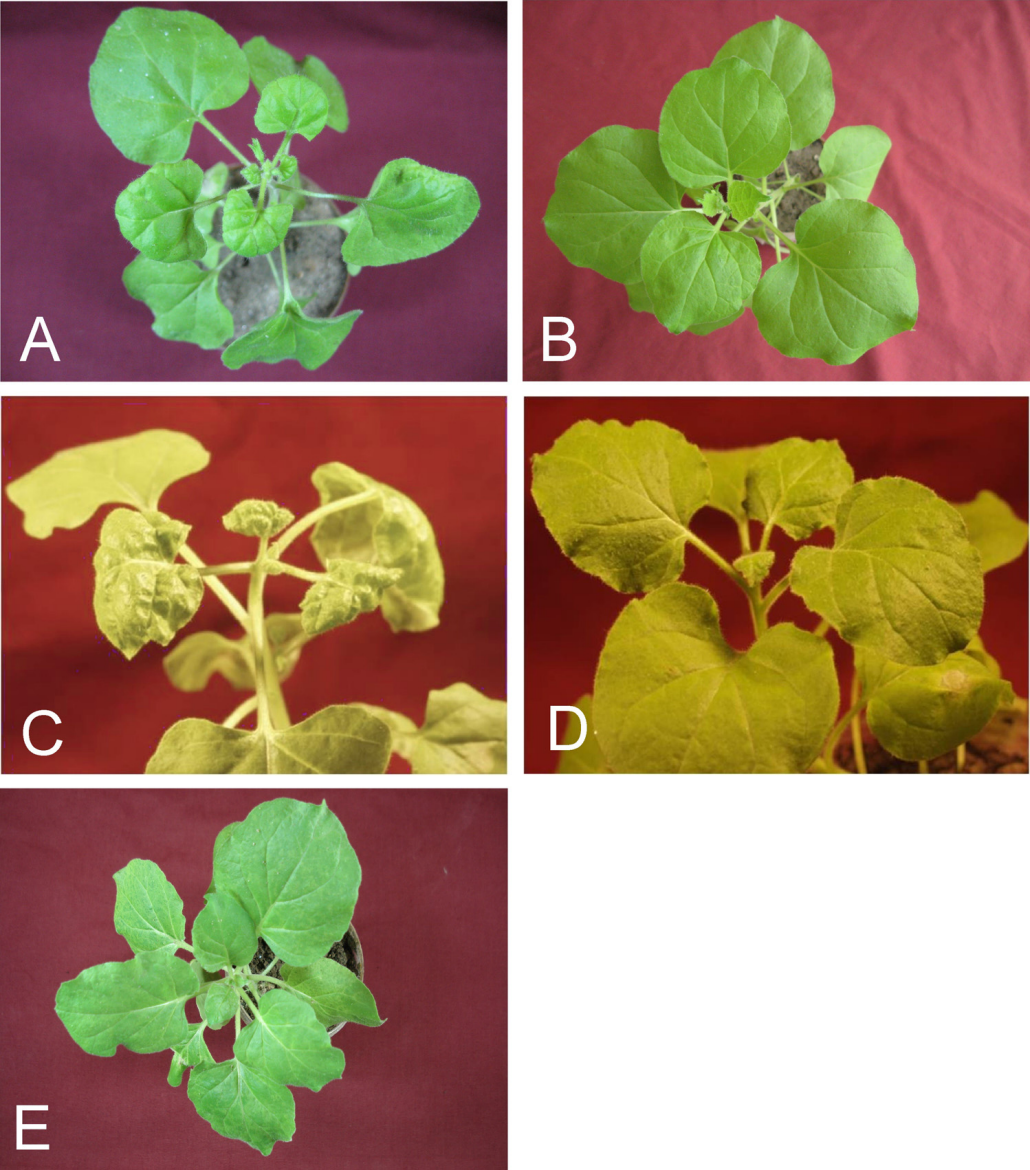
**Transient resistance assays with construct CLCRNAiRepTrAPREn/pFGC in *Nicotiana benthamiana***. Plants were inoculated with CLCuMV (A), CLCuMV and CLCRNAiRepTrAPREn/pFGC (B), CLCuMV and CLCuMB (C) or CLCuMV, CLCuMB and CLCRNAiRepTrAPREn/pFGC (D). A healthy *N. benthamiana *plant is shown for comparison (E). Photographs were taken at 15 days post inoculation.

CLCuMV is infectious to *N. benthamiana *and induces mild symptoms in the absence of the betasatellite but induces severe, CLCuD-like symptoms when co-inoculated with CLCuMB [6,7]. The three hairpin RNAi constructs were co-agroinfiltrated with a partial direct repeat construct of CLCuMV in *N. benthamiana *plants. For each construct 10 plants were used for agroinfiltration. Additionally 15 plants of the same age, grown under the same conditions were inoculated with only CLCuMV. The experiment was repeated three times. All control plants inoculated with only CLCuMV developed typical leaf curl symptoms in systemic leaves, emerging subsequent to inoculation, within 16 days of inoculation. *N. benthamiana *plants co-infiltrated with RNAi constructs and CLCuMV did not exhibit symptoms of infection, even after 45 days (Figure 2A and 2B). This shows that the RNAi constructs targeting different regions of the virus were able to prevent symptomatic infection.

**Figure 3:**
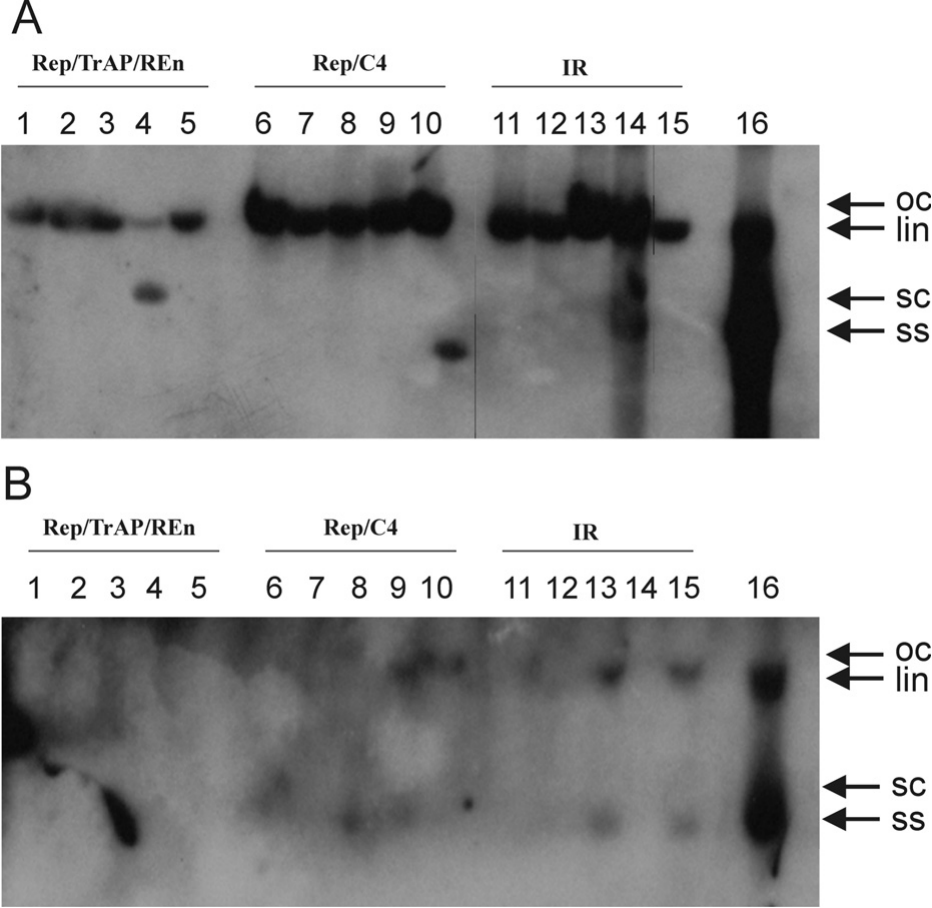
**Southern blot analysis of inoculated *Nicotiana benthamiana *plants**. Southern blots of nucleic acids extracted from the inoculated leaves (panel A) and leaves developing subsequent to inoculation (panel B) were probed with a full-length CLCuMV probe. Samples resulted from plants co inoculated with CLCuMV and either CLCRNAiRepTrAPREn/pFGC (lanes 1 to 5), CLCRNAiRepC4/pFGC (lanes 6 to 10) or CLCRNAiIR/pFGC (lanes 11 to 15). The sample in lane 16 was extracted from a symptomatic plant inoculated with only CLCuMV. The positions of single-stranded (ss), super-coiled (sc), linear (lin) and open-circular (oc) viral DNA forms are indicated with arrows.

Virus replication in inoculated and systemic leaves, which developed subsequent to infiltration, was investigated by Southern blot hybridization. Inoculation of CLCuMV with all three constructs led to a preponderance of linear (lin) and open circular (oc) virus replication forms in the inoculated tissues (Figure 3A). This contrasts with plants inoculated with only CLCuMV in which the single-stranded and supercoiled forms were also detected in significant quantities, which is normal of geminivirus replication. There was a noticeably lower accumulation of lin form for co-inoculation with the CLCRNAiRepTrAPREn/pFGC constructs than the other two constructs. However, in one of the samples analyzed that was co-inoculated with CLCuMV and CLCRNAiRepTrAPREn/pFGC scDNA (Figure 3A, lane 4) was detected with significantly reduced levels of the lin form. Analysis of the systemic leaves, developing subsequent to inoculation, no viral DNA could be detected in plants co-inoculated with the CLCRNAiRepTrAPREn/pFGC, indicating that this construct efficiently prevent spread of the virus from inoculated. For the other two constructs virus replication in systemic leaves was detected in several plants (Figure 3B, lanes 8, 9, 13 and15). However, the levels of virus were significantly less than in a systemic leaf from plant inoculated with only CLCuMV (Figure 3B, lane 16). Thus, only construct CLCRNAiRepTrAPREn/pFGC appeared able to prevent systemic infection whereas the other two constructs reduced the levels of infectivity and, when systemic spread did occur, the amount of virus replication.

### The presence of a betasatellite may compromise RNAi-mediated virus resistance

CLCuMB is essential for the development of *bona fide *disease symptoms in cotton. The satellite encodes an essential pathogenicity determinant that has suppressor of RNA silencing activity [18,19,35]. To investigate whether the presence of a betasatellite may change the outcome of resistance by RNAi the three pFGC5941 constructs were co-agroinfiltrated with constructs for the infectivity of CLCuMV and CLCuMB to *N. benthamiana *plants. The symptoms in control plants, inoculated with only CLCuMV and CLCuMB, appeared within 12-15 days after agroinfiltration. However, co-inoculation of CLCuMV and CLCuMB with CLCRNAiRepC4/pFGC or CLCRNAiIR/pFGC failed to inhibit virus replication and symptoms appeared in all plants within 12 days after agroinfiltration (results not shown). This shows that these two constructs were not effective in controlling CLCuMV in the presence of CLCuMB. In contrast, for co-inoculation with CLCRNAiRepTrAPREn/pFGC, only 2 out of 10 plants inoculated developed symptoms at 20 days after inoculation and the remainder of the plants remained asymptomatic, thus showing the silencing efficiency of the construct even in the presence of CLCuMB (Figure 2).

### Transient resistance assays with PVX expressing Rep protein of heterologous begomoviruses

In investigate whether the CLCRNAiRepTrAPREn/pFGC RNAi construct containing sequences derived from CLCuMV would provide resistance against heterologous viruses, the construct was co-inoculated with a PVX vector expressing the Rep genes of *Cabbage leaf curl virus *(CabLCV) or *African cassava mosaic virus *(ACMV); two begomoviruses only distantly related to CLCuMV whose Rep genes show 82% and 86% nucleotide sequence identity, respectively, to the Rep gene of CLCuMV.

**Figure 4:**
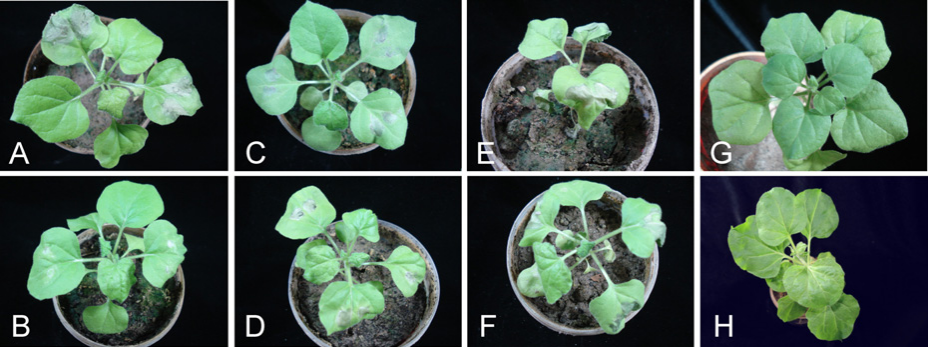
**Transient resistance assays with PVX expressing the Rep genes of heterologous viruses in *Nicotiana benthamiana***. Plants were inoculated with PVX/CLCuMV-Rep (panel A), PVX/CLCuMV-Rep and CLCRNAiRepTrAPREn/pFGC (panel B), PVX/CabLCuV-Rep (panel C), PVX/CabLCuV-Rep and CLCRNAiRepTrAPREn/pFGC (panel D), PVX/ACMV-Rep (panel E) or PVX/ACMV-Rep and CLCRNAiRepTrAPREn/pFGC (panel F). Panels G and H show a *N. benthamiana *plant inoculated with the PVX vector lacking an insert and a healthy, non-inoculated *N. benthamiana *plant, respectively. Photographs were taken at 15 days post inoculation.

Inoculation of *N. benthamiana *plants with PVX expressing the Rep genes of CLCuMV (PVX/CLCuMV-Rep) and ACMV (PVX/ACMV-Rep) induced severe necrosis of inoculated tissues (Figure 4 panels A and E, respectively), whereas the necrosis induced by PVX expressing the CabLCuV Rep gene (PVX/CabLCuV-Rep) induced a somewhat milder necrosis (Figure 4 panel C). Symptoms of systemic infection appeared on leaves developing subsequent to inoculation at 10-15 days post-inoculation. This consisted of leaf curl and necrosis for all three PVX constructs, although the symptoms were milder for the CabLCuV Rep than the other two Rep expressing PVX constructs (results not shown).

Co-inoculation of PVX/CLCuMV-Rep with CLCRNAiRepTrAPREn/pFGC did not lead to necrosis of the inoculated tissue. In contrast, inoculation of CLCRNAiRepTrAPREn/pFGC with either PVX/ACMV-Rep or PVX/CabLCuV-Rep did lead to necrosis of inoculated tissues (Figure 4 panels F and D, respectively). However, for all three PVV constructs, co-inoculation with CLCRNAiRepTrAPREn/pFGC did not ultimately prevent systemic infection and symptoms appearing on leaves developing subsequent to inoculation.

## Discussion

Although natural host-plant resistance remains the most desirable and easily introduced means of reducing losses to phytopathogenic viruses, for geminiviruses the lack of suitable genetic sources of resistance (germplasm) in many cases means that this is not an option. This is unfortunately the case for cotton in Pakistan, which is the main foreign exchange earner of the country, where an epidemic of CLCuD in the 1990 s led to massive losses [1]. Host-plant resistance introgressed into commercial cotton during the 1990 s [36] was rapidly overcome by a resistance breaking strain [25] and all commercial/cultivated cotton varieties available at this time are susceptible [37]. There is thus the need for alternative sources of resistance which can complement host plant resistance.

The study here was initiated to determine which sequences of the genome of a monopartite begomovirus are the most efficient at delivering RNAi-mediated resistance and what effects the presence of a betasatellite might have on the outcome of RNAi-mediated resistance. The results show that, although all three constructs spanning the complementary-sense sequences of the genome are able to prevent symptoms in *N. benthamiana *by CLCuMV, only one (CLCRNAiRepTrAPREn/pFGC) was able prevent systemic infection. The reason for the superior performance of the CLCRNAiRepTrAPREn/pFGC construct is possibly that it spans three virus-encoded genes; Rep and REn, which are important for viral DNA replication [38,39], and TrAP. The begomovirus-encoded TrAP is a multifunctional protein that plays an important role in host-virus interactions. It is a transcription factor required for the expression of late (virion-sense) genes for bipartite begomoviruses [40,41], can be a pathogenicity factor [42], may counter programmed cell death [43], conditions a virus-nonspecific enhanced-susceptibility phenotype in transgenic plants [44], interacts with and inactivates SNF1-related kinase [45] and may also suppress RNAi [46], possibly by inhibiting adenosine kinase [47,48].

The results obtained here thus show that not all virus derived sequences will deliver efficient silencing mediated resistance to begomoviruses. A similar conclusion was reached for RNAi-mediated resistance to TSWV where only expression of either the N or NS_m _gene sequences yielded resistance [49]. Our results suggest that the sequences of the TrAP gene are the most efficient for delivering resistance, possibly because the TrAP is a suppressor of silencing and down-regulation of expression of this protein compromises the virus' ability infect plants.

Although the CLCRNAiRepTrAPREn/pFGC construct was able to prevent symptomatic infection of CLCuMV, in the presence of CLCuMB a significant proportion of plants exhibited symptoms. This suggests that the betasatellite may compromise the resistance. Betasatellites encode a single protein, βC1, which is a dominant pathogenicity (symptom) determinant which also has suppressor of RNAi activity [18,36,50]. Thus the additional suppressor activity and dominant symptom determinant provided by the betasatellite, not targeted by sequences contained in CLCRNAiRepTrAPREn/pFGC, may be able to overcome the silencing by the construct.

A major drawback of RNA-mediated resistance, being sequence-based, is its high degree of specificity. Thus the resistance will only be functional against viruses with very similar sequences across the targeted region. For example, transgenically expressed sequences of the tospovirus *Tomato spotted wilt virus *(TSWV) provide protection against the homologous virus but not against the related viruses *Groundnut ring spot virus *and *Tomato chlorotic spot virus*, despite the fact that they share 78 to 80% nucleotide sequence identity across their genomes [49]. This is consistent with the results obtained here. The CLCuMV derived construct CLCRNAiRepTrAPREn/pFGC efficiently prevented Rep induced necrosis of inoculated tissues for the PVX vector expressing CLCuMV Rep but not the PVX vectors expressing the Rep genes of the distantly related begomoviruses ACMV and CabLCuV.

The ultimate aim of this work is to develop a broad spectrum virus-resistance that can be deployed in a number of crops but particularly in cotton. Efforts are now underway to assess whether the results with the CLCRNAiRepTrAPREn/pFGC construct results can be reproduced in transgenic plants and particularly whether the betasatellite is going to be a problem for RNAi-mediated resistance to begomoviruses.

## Competing interests

The authors declare that they have no competing interests.

## Authors' contributions

MM and MH conducted all the experiments and wrote the paper. SM and RWB edited the paper and coordinated the research efforts. All authors have read and approved the manuscript.
